# Diagnostic Challenges and Modern Therapeutic Strategies in Giant Cell Arteritis

**DOI:** 10.3390/diagnostics16030470

**Published:** 2026-02-03

**Authors:** Alicia Rodriguez-Pla

**Affiliations:** Premier Rheumatology, P. C., 1189 E Herndon Avenue, Suite 101, Fresno, CA 93720, USA; arp@premierrheumatology.org; Tel.: +1-559-421-3768

**Keywords:** giant cell arteritis, temporal arteritis, vasculitis, diagnostic challenges, differential diagnosis, temporal artery biopsy

## Abstract

Giant cell arteritis (GCA) represents one of the most diagnostically challenging systemic vasculitides, characterized by its heterogeneous clinical presentation, lack of pathognomonic features, and potential for devastating complications, with a special concern for irreversible vision loss. This comprehensive review synthesizes current evidence regarding the multifaceted diagnostic challenges in GCA, incorporating recent advances in classification criteria, imaging technologies, biomarker research, and emerging therapeutic strategies.

## 1. Introduction and Clinical Significance

GCA is an inflammatory disease of large and medium-sized vessels that predominantly affects individuals over 50 years of age, with peak incidence between 70 and 80 years [1], and it is specially concerning for visual loss [2]. The condition’s clinical significance extends beyond its inflammatory nature, as delayed diagnosis can result in catastrophic complications, including permanent blindness, which occurs in 15–20% of untreated patients, stroke, aortic dissection, and aortic aneurysm formation [3,4,5].

The pathophysiology of GCA involves complex interactions between adaptive and innate immune systems, with granulomatous inflammation involving arterial walls and resulting in vessel occlusion and tissue ischemia [6]

**Figure 1:**
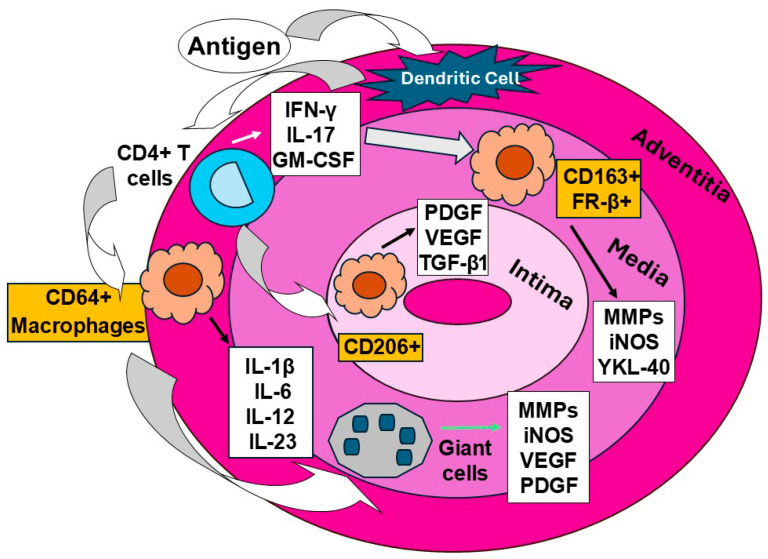
**Compartmentalized macrophage and T cell effector functions in GCA: layer-specific immunopathology and steroid resistance mechanisms.** The inflammatory landscape of GCA exhibits a functionally distinct “division of labor” across the arterial wall, with each compartment contributing to specific pathogenic outcomes. (A) Adventitia, systemic signaling hub: CD64^+^ macrophages and CD4^+^ Th17 cells orchestrate systemic inflammation through production of IL-6, IL-1β, and IL-23, which drive hepatic acute-phase protein synthesis, with elevated CRP and serum amyloid A (SAA), and constitutional symptoms like fever and malaise. This axis is highly glucocorticoid-responsive. (B) Media, zone of vascular wall destruction: multinucleated giant cells derived from macrophage fusion and CD206^+^ alternatively activate macrophages localize to the internal elastic lamina (IEL), where they secrete MMP-9, MMP-2, and the chitinase 3-like protein YKL-40. These proteases, combined with reactive oxygen species (ROS) by activated nicotinamide adenine dinucleotide phosphate (NADPH) oxidase, mediate degradation of elastin and collagen, resulting in focal wall thinning, medial necrosis, and aneurysmal dilatation. (C) Intima, zone of occlusive remodeling: CD163^+^/FR-β^+^ alternatively activate macrophages, and resident myofibroblasts secrete platelet-derived growth factor (PDGF) and vascular endothelial growth factor (VEGF), promoting neoangiogenesis, intimal smooth muscle cell hyperplasia, and progressive luminal narrowing, leading to tissue ischemia. While the adventitial IL-6/Th17 axis responds dramatically to glucocorticoids, the medial giant-cell-driven matrix destruction and CD163^+^-mediated intimal remodeling exhibit relative steroid resistance, perpetuating subclinical inflammation and late-stage ischemic complications even after initial clinical remission. Abbreviations: CD, cluster of differentiation; iNOS, inducible nitric oxide synthase; MMP: matrix metalloproteinases; PDGF, platelet-derived growth factor; IL: interleukin; GM-CSF: granulocyte macrophage colony-stimulating factor; TGF-β1, transforming growth factor-β1; IFN, interferon; FR, folate receptor.

The reason GCA is so difficult to manage is that different “pockets” of inflammation in the artery respond differently to therapy. The clinical manifestations of GCA are the direct result of a spatially organized inflammatory process within the temporal artery wall. Recent research using spatial transcriptomics has demonstrated that macrophages and T cells are not uniformly distributed; rather, they organize into three functionally distinct layers that dictate disease progression and treatment response [7,8]. The adventitia serves as the primary gateway for leukocyte entry and the epicenter of the systemic acute-phase response. Here, a cluster of differentiation (CD)64^+^ macrophages and Th17 cells forms a potent proinflammatory circuit. This layer is characterized by the high production of interleukin (IL)-6, IL-1β, and IL-23. This “adventitial signature” correlates strongly with elevated C-reactive protein (CRP), erythrocyte sedimentation rate (ESR), and constitutional symptoms such as fever and weight loss. Importantly, this pathway is highly sensitive to glucocorticoids and IL-6 receptor antagonists, e.g., tocilizumab, often resulting in a rapid normalization of systemic markers. Tissue injury is concentrated in the arterial media, where multinucleated giant cells and CD206^+^ macrophages orchestrate the degradation of the internal elastic lamina. This destructive phenotype is driven by the secretion of matrix metalloproteinase (MMP)-9, YKL-40/chitinase 3-like, and reactive oxygen species (ROS). Recent data highlight granulocyte macrophage colony-stimulating factor (GM-CSF) as the master regulator of this medial destruction, providing a rationale for the use of GM-CSF receptor blockers, e.g., mavrilimumab, to prevent permanent structural damage to the vessel wall. The most clinically catastrophic feature of GCA, luminal occlusion, occurs in the intima. This layer is dominated by CD163^+^ and folate receptor (FR)-β+ macrophages, which drive a “response-to-injury” program. These cells secrete vascular endothelial growth factor (VEGF) and platelet-derived growth factor (PDGF), triggering neoangiogenesis and the migration of myofibroblasts. Unlike the adventitial inflammatory markers, this intimal remodeling program is notably resistant to glucocorticoids. This explains the “vascular-systemic dissociation” frequently observed in GCA, where patients may develop ischemic complications, e.g., vision loss, despite having a normal CRP and an apparently suppressed systemic inflammatory response (Figure 1) [7,8,9,10,11,12].

Despite significant advances in understanding pathogenic mechanisms, the etiology remains unknown, and diagnostic approaches continue to rely on combinations of clinical assessment, laboratory findings, imaging, and histopathological examination [13].

The diagnostic challenges of GCA can be divided into two major categories: (1) failure to diagnose the disease correctly and in a timely manner, which may be complicated due to its unspecific symptoms; (2) diagnosing GCA in patients who do not have the disease but a mimicker.

## 2. Material and Methods

This narrative review summarizes current evidence on diagnostic challenges, including evolving classification criteria and therapeutic strategies in GCA. We conducted a comprehensive literature search of PubMed/MEDLINE, Web of Science, and Scopus databases for articles published through December 2025. Search terms included combinations of MeSH terms and keywords: “giant cell arteritis”, “temporal arteritis”, “large vessel vasculitis”, “diagnosis”, “classification criteria”, “imaging”, “ultrasound”, “temporal artery biopsy”, “biomarkers”, “treatment”, “glucocorticoids”, and “biologics”.

We prioritized peer-reviewed original research articles, systematic reviews and meta-analyses, randomized controlled trials, large observational cohort studies, and evidence-based clinical guidelines. We primarily focused on publications from the last 10 years while including seminal earlier works that established foundational concepts. We focused on clinically relevant studies addressing diagnostic accuracy, classification criteria performance, imaging modality comparisons, biomarker validation, and therapeutic efficacy.

We emphasize that this is narrative and not a systematic review. Therefore, we did not conduct formal systematic quality assessments or meta-analysis, but we critically evaluated the strength and consistency of evidence throughout our synthesis. By “prioritization”, we mean that when multiple sources addressed the same topic, we preferentially cited peer-reviewed original research, systematic reviews, meta-analyses, and randomized controlled trials over case reports, editorials, or lower-quality evidence. This is a narrative synthesis based on expert clinical judgment rather than a systematic review with predefined inclusion criteria and quality scoring. Throughout the manuscript, we explicitly note areas of conflicting evidence, methodological limitations of studies, and gaps in the current literature to provide appropriate critical appraisal while maintaining the narrative review format.

## 3. Risk Factors and Epidemiological Consideration

### 3.1. Genetic Factors

GCA is not considered a purely hereditary disease that is directly inherited. Research indicates that a genetic predisposition plays a role in increasing an individual’s susceptibility to GCA. However, although certain genetic factors may raise the risk, they do not guarantee the development of the disease.

Genetic predisposition is suggested through familial clustering and association with specific human leukocyte antigen (HLA) alleles, though the specific genetic factors remain unidentified. The HLA-DRB1*04 alleles, especially HLA-DRB10*401 and HLA-DRB10*404, have been identified as significant genetic risk factors, particularly in Caucasian populations. The HLA-DRB1 molecules are involved in antigen presentation to CD4+ T cells, suggesting that specific HLA variants may present antigens that trigger pathogenic immune responses in genetically predisposed individuals. While there is a higher incidence of GCA in families with a history of the condition, familial cases are relatively rare, accounting for about 1% of all patients. This complex genetic landscape underscores that while genetics contribute to risk, there are other factors also at play [14,15].

### 3.2. Geographic and Demographic Patterns

The incidence of GCA varies significantly worldwide, with North American estimates ranging from 15 to 30 cases per 100,000 individuals aged 50 and older [16]. Scandinavian countries report the highest incidence rates, suggesting that genetic, environmental, and/or geographic factors may play important roles in disease susceptibility [17]. A recent meta-analysis examining nine studies found an overall pooled prevalence of 51.74 cases per 100,000 people over 50 years, with prevalence remaining stable across publication years using linear fit modeling [18].

The female predominance is consistent across populations, with women affected 2–4 times more frequently than men [16]. This gender disparity likely reflects hormonal influences on immune system function, though the specific mechanisms remain incompletely understood [19]. Age represents the most significant risk factor, with the disease rarely occurring before age 50 and demonstrating an exponential increase in incidence with advancing age [1,20].

Some studies reported a seasonal variation with increased diagnostic frequency during fall and winter months, suggesting possible infectious triggers or environmental precipitants [21], although none of them has been yet demonstrated.

### 3.3. Pathophysiological Risk Factors

Several age-related biological processes contribute to GCA susceptibility. Immunosenescence, characterized by declining naïve T and regulatory T cell populations, accumulation of CD28-null T cells with enhanced cytotoxic potential, decreased thymic output reducing T cell receptor diversity, and increased proinflammatory cytokine production, including IL6-IL1β, and antitumor necrosis factor (TNF)α, which creates a proinflammatory state termed “inflammaging”, and shifts in CD4/CD8 cell distributions, creates a proinflammatory milieu conducive to vasculitis development [10,22,23].

Concurrent vascular aging, marked by loss of arterial elasticity and increased wall stiffness, provides the structural substrate for inflammatory targeting. Age-related loss of adventitial *vasa vasorum* may create hypoxic conditions in the arterial wall, while accumulation of oxidative damage and advanced glycation end products in the elastic lamina may generate neoantigens that trigger autoimmune responses [24,25].

## 4. Clinical Presentation and Diagnostic Complexity

### 4.1. Cranial Manifestations

The classic cranial presentation of GCA encompasses a constellation of symptoms that, while characteristic, are not pathognomonic. New-onset headaches are often described as severe and different from previous headache patterns [26,27,28]. Temporal artery abnormalities, including tenderness, thickening, or reduced pulsation, provide important physical examination findings and are present in 40–60% of cases, making them one of the more common physical signs in GCA [13,29].

Jaw claudication due to ischemia of masticatory muscles during chewing demonstrates high specificity for GCA, approximately 90%, and occurs in 35–50% of patients [30,31,32]. While jaw claudication is less common than temporal artery abnormalities, its high specificity makes it a particularly valuable diagnostic clue when present [13,33,34]. The “chewing gum test”, while not superior to clinical history alone, may provide additional diagnostic utility when pain onset occurs within two minutes of chewing [35].

Visual complications are also part of the cranial manifestations, but we will discuss them separately given their potentially devastating clinical consequences.

### 4.2. Visual Complications

Visual manifestations represent the most feared complications of GCA, occurring in 15–20% of patients and potentially progressing to irreversible blindness [36,37,38]. Arteritic anterior ischemic optic neuropathy (AAION) constitutes the most common mechanism, characterized by acute monocular vision loss with “chalk-white” optic disc edema, often accompanied by retinal whitening or cotton wool spots [39,40].

The spectrum of visual involvement includes transient visual disturbances, diplopia from cranial nerve palsies, and various patterns of visual field defects [41,42]. Central retinal artery occlusion occurs in approximately 10% of cases [43,44], while posterior ischemic optic neuropathy and choroidal infarction represent less common but serious manifestations [45,46,47,48]. The irreversible nature of most visual complications underscores the critical importance of rapid diagnosis and treatment initiation [49,50].

### 4.3. Systemic and Constitutional Symptoms

Constitutional symptoms, including fatigue, fever, anorexia, and unintentional weight loss, occur in 40–60% of GCA patients [51,52,53]. These non-specific symptoms often precede more characteristic manifestations and may lead to diagnostic delays, particularly in patients presenting with isolated systemic features [53,54].

Polymyalgia rheumatica (PMR) coexists with GCA in approximately 40–50% of cases, manifesting as bilateral shoulder and pelvic girdle pain, weakness, and stiffness [55]. The relationship between PMR and GCA remains complex, with some experts considering them manifestations of the same disease spectrum [56]. PMR symptoms may precede, accompany, or follow GCA diagnosis, and their presence should prompt careful evaluation for underlying vasculitis [57].

### 4.4. Extracranial Large Vessel Disease

Recognition of extracranial large vessel involvement has expanded significantly with advanced imaging capabilities. Large vessel GCA (LV-GCA) affects the aorta and its major branches in 50–80% of patients, depending on imaging modality sensitivity [58]. This phenotype may be present without classic cranial symptoms, leading to diagnostic challenges and potential delays [59,60,61]. Patients with isolated extracranial disease often present with non-specific constitutional symptoms, refractory PMR, fever of unknown origin, or limb claudication. Specific presentations may include limb claudication, upper extremities more commonly than lower, blood pressure discrepancies > 10 mmHg between arms in cases of subclavian involvement, carotid or subclavian bruits, or absent pulses, especially radial pulses. Some patients report unexplained fever, night sweats, or refractory PMR that fails to respond adequately to moderate-dose glucocorticoids.

Characteristic imaging findings in LV-GCA include concentric mural thickening with fluorodeoxyglucose (FDG) uptake or contrast enhancement in a “macaroni sign” pattern, typically involving subclavian arteries (75–80%), thoracic aorta (40–60%), and abdominal aorta (30–40%). The axillary arteries show bilateral involvement in a majority of cases, which is highly specific for GCA versus atherosclerotic disease [62].

The absence of temporal artery abnormalities in these patients necessitates high clinical suspicion and appropriate imaging to establish diagnosis. Recognition of isolated LV-GCA is critical as these patients often do not meet traditional diagnostic criteria focused on cranial symptoms, potentially leading to diagnostic delay. The 2022 American College of Rheumatology (ACR)/European League Against Rheumatism (EULAR) criteria partially address this gap by incorporating imaging of large vessels and assigning points for bilateral axillary involvement [63,64].

Patients with extensive large vessel involvement may require longer duration of glucocorticoid therapy and have higher relapse rates during tapering. Some evidence suggests that these patients may benefit from earlier introduction of steroid-sparing agents, though this remains an area requiring further study [65].

## 5. Laboratory Findings and Biomarkers

### 5.1. Traditional Inflammatory Markers

Erythrocyte sedimentation rate (ESR) and C-reactive protein (CRP) remain cornerstone laboratory tests in GCA evaluation, though their limitations are well recognized. ESR elevation (>50 mm/h using the Westergren method) occurs in approximately 76–84% of GCA patients, while CRP demonstrates slightly higher sensitivity, at 86–98%, depending on the study population [66,67,68,69,70]. However, normal inflammatory markers occur in approximately 4–20% of biopsy-proven cases (with most studies reporting 7–10%), representing a population enriched for atypical presentations such as early disease course, predominantly large vessel involvement without cranial manifestations, or concurrent use of medications that suppress inflammatory markers such as tocilizumab or glucocorticoids [59,71,72].

The combination of ESR and CRP provide enhanced diagnostic utility, with concordant elevation yielding 99% sensitivity for positive temporal artery biopsy. Conversely, both normal ESR and CRP confer high negative predictive value, though this cannot definitively exclude diagnosis in patients with high clinical suspicion [70].

### 5.2. Platelet Count and Hematological Parameter

Thrombocytosis (>400 × 10^3^/μL) occurs in 50–70% of GCA patients (range from multiple studies) and demonstrates comparable diagnostic performance to traditional inflammatory markers [73]. Platelet counts >400 × 10^3^/μL show specificity of 80–90% (95% confidence interval (CI): 75–92%) for positive temporal artery biopsy, though sensitivity remains moderate at 50–60% [74]. The combination of elevated CRP and platelet count may provide optimal diagnostic utility, with both positive tests yielding 77% positive predictive value [75].

Normochromic normocytic anemia affects approximately 60–80% of patients, with mean hemoglobin levels around 11.7 ± 1.6 g/dL [76]. Leukocytosis occurs less consistently but may accompany active disease phases [77].

### 5.3. Emerging Biomarkers

Research into novel biomarkers has identified several promising candidates that could potentially enhance diagnostic accuracy and disease monitoring. However, while numerous candidate biomarkers have been identified, most have been evaluated in single-center studies with limited sample sizes and lack independent validation in diverse populations. Furthermore, few studies have demonstrated that these biomarkers provide diagnostic or prognostic information beyond that available from ESR, CRP, and platelet count.

Among emerging biomarkers, SAA shows the most clinical potential because it strongly correlates with disease activity, it demonstrates elevation in GCA patients compared to healthy controls, and it demonstrates superior sensitivity to ESR for detecting active vasculitis (85–90% vs. 76–84%). SAA may decline more rapidly with effective treatment, potentially enabling earlier detection of inadequate response [78,79,80]. SAA assays are already available in many clinical laboratories, facilitating rapid translation. However, SAA lacks specificity for GCA vs. other inflammatory conditions, as in the case of ESR and CRP [78].

Cytokine profiling reveals elevated levels of interleukin-6 (IL-6), IL-23, and various chemokines in GCA patients [81,82,83,84]. IL-6 levels correlate with disease activity and form the rationale for tocilizumab therapy [81]. B-cell activating factor (BAFF) and chitinase 3-like protein (YKL-40) show promise as inflammatory markers with potential roles in disease pathogenesis [85,86].

Vascular remodeling biomarkers including matrix metalloproteinases (MMPs), especially MMP-2, MMP-9, and MMP12; vascular endothelial growth factor (VEGF); and angiopoietins may provide insights into vessel wall damage and repair processes [11,12,15,87]. These reflect vascular remodeling processes but are not GCA-specific, and require specialized assays not widely available. These may have greater utility as research tools for understanding pathogenesis than as clinical biomarkers.

The most promising future approach may be composite biomarker panels combining traditional markers (ESR, CRP, platelets) with selected novel markers (e.g., SAA, IL-6) and clinical parameters, potentially enhanced by machine learning algorithms. Such algorithms could improve diagnostic accuracy and predict relapse risk but require rigorous validation in prospective multicenter cohorts before clinical implementation. It is important to take into account that none of these novel biomarkers have achieved clinical implementation yet, and further validation studies are required.

## 6. Imaging Advances and Diagnostic Modalities

### 6.1. Ultrasound as First-Line Imaging

Color Doppler ultrasound (CDUS) has emerged as the preferred initial imaging modality for suspected GCA with cranial involvement, as supported by 2018 EULAR recommendations [88]. The pathognomonic “halo sign”, representing hypoechoic vessel wall thickening, demonstrates sensitivity of 43–77% and specificity of 89–100% when compared to ACR 1990 criteria [89]. However, while the 2018 EULAR recommendations support CDUS as first-line imaging for suspected cranial GCA, implementation varies globally due to limited availability of trained operators experienced in vascular ultrasound, particularly in resource-limited settings, rural areas, and healthcare systems without dedicated rheumatology-based ultrasound programs [90]. Ultrasound advantages include non-invasiveness, immediate availability when integrated into rheumatology practice, cost-effectiveness, and ability to examine multiple vessel territories [91,92]. The technique allows assessment of both cranial arteries (temporal, occipital, facial) and extracranial vessels (axillary, subclavian, carotid) [93]. Bilateral halo signs increase diagnostic specificity to 100%, though sensitivity decreases with glucocorticoid treatment duration [94].

Technical considerations for optimal ultrasound performance include the use of high-frequency transducers (>15 MHz), experienced sonographers, and standardized examination protocols [95]. The addition of extracranial vessel assessment increases sensitivity from 70% to 89% while maintaining specificity at around 91% [89,92].

### 6.2. Advanced Cross-Sectional Imaging

Magnetic resonance imaging (MRI) with vessel wall imaging has demonstrated excellent diagnostic performance, with sensitivity of 94% and specificity of 85% for GCA diagnosis. High-resolution MRI can detect mural inflammation in temporal arteries, with grading systems distinguishing physiologic from pathologic enhancement. The technique shows utility in extracranial vessel assessment and may obviate need for temporal artery biopsy when clearly normal [96,97].

Computed tomography angiography (CTA) provides complementary structural information regarding vessel stenosis, occlusion, and aneurysm formation [98,99,100]. While less sensitive than MRI for early inflammatory changes, CTA offers superior assessment of calcification and luminal abnormalities [100].

### 6.3. Nuclear Medicine Imaging

18F-fluorodeoxyglucose (FDG) positron emission tomography (PET)/computerized tomography (CT) demonstrates sensitivity of 80% and specificity of 91% for large vessel vasculitis diagnosis [101]. The technique excels in detecting aortic and major branch vessel involvement, with particular utility in extracranial disease phenotypes [102]. FDG uptake typically affects subclavian arteries (75%), thoracic and abdominal aorta (50%), and axillary arteries (40%) [103].

PET/CT limitations include reduced sensitivity in cranial vessels due to spatial resolution constraints and decreased uptake following glucocorticoid treatment initiation [104]. The technique requires careful timing, with optimal diagnostic yield within three days of treatment and declining sensitivity after 10 days [105].

### 6.4. Multimodal Imaging Approaches

Integration of multiple imaging modalities provides comprehensive vascular assessment and may optimize diagnostic accuracy [106]. Proposed algorithms suggest initiating with ultrasound for cranial assessment, followed by FDG-PET/CT or MRI for extracranial evaluation in appropriate clinical contexts. This approach maximizes detection of both cranial and large vessel involvement while maintaining cost-effectiveness [107,108].

### 6.5. Fast-Track Diagnostic Pathways

Fast-track clinics dedicated to rapid evaluation of suspected GCA have demonstrated significant improvements in patient outcomes. These pathways typically incorporate same-day or next-day assessment combining clinical evaluation, inflammatory marker testing, and vascular ultrasound, with treatment initiation based on this integrated assessment.

Evidence from multiple centers demonstrates that fast-track pathways reduce time to diagnosis from weeks to days (median 0–2 days vs. 9–24 days in traditional pathways), decrease rates of irreversible vision loss (0–5% vs. 13–20%), and reduce unnecessary temporal artery biopsies and prolonged glucocorticoid courses in patients without GCA [109,110]. Cost-effectiveness analyses suggest that these pathways are economically favorable due to prevention of vision loss and reduced diagnostic testing.

Critical elements of successful fast-track clinics include dedicated access for urgent referrals, availability of experienced vascular ultrasonographers, integrated rheumatology–ophthalmology care pathways for patients with visual symptoms, and standardized protocols for treatment initiation and follow-up. Implementation challenges include resource allocation, staff training, and establishing referral networks, but outcomes support wider adoption of this model.

### 6.6. Integration of 2023 EULAR Imaging Recommendations

The 2023 EULAR recommendations for imaging in large vessel vasculitis provide updated guidance emphasizing multimodal imaging approaches. Key recommendations include the following: (1) CDUS as first-line imaging for suspected cranial GCA when expertise is available; (2) FDG-PET/CT, MRA, or CTA for assessment of extracranial large vessel involvement; (3) systematic evaluation of both cranial and extracranial vessels in all GCA patients when feasible; (4) recognition that negative imaging does not exclude GCA in patients with high clinical suspicion; and (5) consideration of repeat imaging in patients with disease relapse or atypical features. These recommendations emphasize that imaging should complement, rather than replace, clinical judgment and that the choice of modality should consider local expertise, availability, and individual patient factors [107].

## 7. Temporal Artery Biopsy: Evolving Role

### 7.1. Current Diagnostic Performance

**Figure 2:**
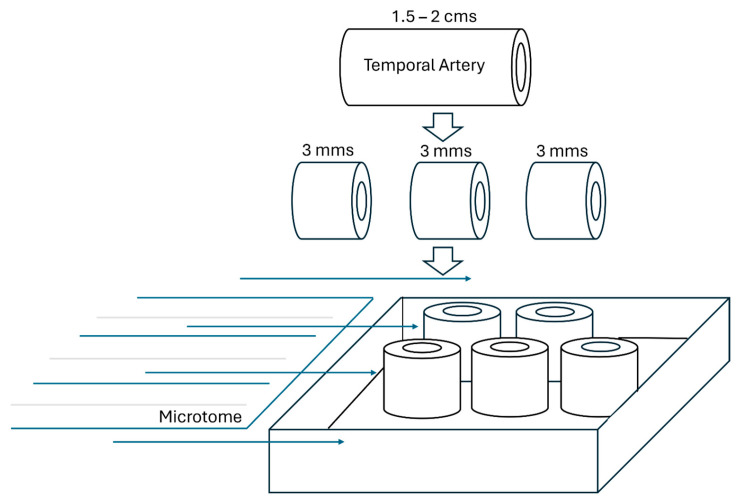
**Schematic representation of pathological processing of temporal artery specimens.** To minimize the high rate of false-negative results due to “skip lesions” (areas of inflammation interspersed with normal artery) or inadequate sampling, the guidelines standardize the handling of the specimen. (1) Optimal specimen length: the consensus recommends an adequate length (often cited as 1.5–2 cm to maximize the chance of capturing a skip lesion. (2) Complete submission: The entire segment of the biopsied artery should be submitted for processing. (3) Serial sectioning: Instead of only examining a few cuts, the specimen should be evaluated by serial sectioning at multiple levels. This means cutting the entire artery segment into numerous thin sections (e.g., in a specific interval) to examine the vessel at many different points along its length, drastically improving the chance of finding a focal lesion.

The temporal artery biopsy (TAB) remains important as a diagnostic tool despite imaging advances, with specificity approaching 100% and sensitivity of 61–77% [111]. The Society for Cardiovascular Pathology guidelines emphasize standardized processing and interpretation protocols to optimize diagnostic yield [112] (Figure 2).

Factors affecting TAB sensitivity include specimen length (optimal >2 cm), number of examined sections, timing relative to glucocorticoid initiation, and disease segmental nature [113]. Bilateral biopsy may improve diagnostic yield in cases of high clinical suspicion with negative unilateral results [111].

### 7.2. Histopathological Considerations

**Figure 3:**
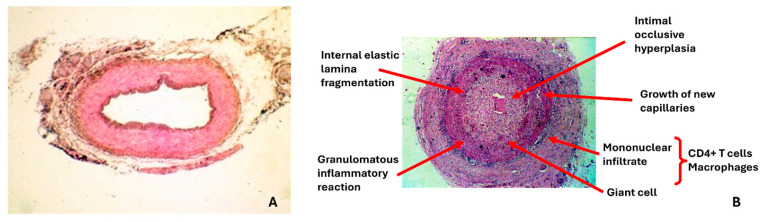
(**A**) **Image of a normal temporal artery biopsy without signs of inflammation/vasculitis.** Hematoxylin and eosin (H&E) stain, original magnification ×25. (**B**) **This image demonstrates findings consistent with vasculitis in a temporal artery biopsy, a common finding in GCA.** Key features shown include the following. (1) Panarteritis: A dense inflammatory infiltrate of mononuclear cells is visible throughout all layers of the vessel wall (tunica adventitia, media, and intima). (2) Intimal thickening and lumen occlusion: The inner layer (tunica intima) is markedly thickened and proliferative, leading to severe luminal stenosis and near-occlusion of the artery’s central channel. (3) Thrombus formation: Fibrinoid material and inflammatory cells are seen within the center of the narrowed lumen. Hematoxylin and eosin (H&E) stain, original magnification ×25 (same magnification as panel A for direct comparison). Temporal artery biopsy specimens are from the personal collection of Dr. Rodriguez-Pla.

Characteristic histopathological features include granulomatous inflammation with multinucleated giant cells, IEL destruction, and arterial wall thickening [112] (Figure 3). However, giant cells are present in only 50–60% of positive biopsies, and their absence does not exclude diagnosis [113]. Alternative inflammatory patterns, including lymphocytic infiltration without giant cells, may represent valid diagnostic findings [113,114].

The distinction between “healed arteritis” and age-related arteriosclerosis affecting the temporal artery remains challenging and lacks definitive criteria [115]. Recent guidelines recommend caution in diagnosing healed arteritis based solely on intimal thickening or medial fibrosis without clear inflammatory markers [112].

### 7.3. Integration with Clinical Assessment

TAB should be considered within comprehensive clinical context rather than as a standalone diagnostic test [116]. The procedure remains valuable when imaging results are inconclusive or contraindicated, and in research settings requiring histopathological confirmation [13,117]. Fast-track clinical pathways often initiate treatment based on clinical and imaging findings while awaiting biopsy results [109].

## 8. Classification Criteria Evolution

### 8.1. 1990 American College of Rheumatology (ACR) Criteria Limitations

The 1990 ACR classification criteria require three of five features: age > 50 years, new headache, temporal artery abnormality, ESR > 50 mm/h, and abnormal arterial biopsy [118]. These criteria demonstrate 93.5% sensitivity and 91.2% specificity when distinguishing GCA from other forms of vasculitis in patients already diagnosed with vasculitis (original ACR validation study [118]), but show significantly lower sensitivity (53–80%) when applied in broader clinical settings where the differential diagnosis included non-vasculitic conditions. These criteria show limitations in contemporary practice.

Recognized shortcomings include underrepresentation of patients with isolated ocular manifestations, lack of incorporation of imaging findings, absence of large vessel involvement criteria, and potential for missing cases with normal inflammatory markers [119]. The criteria were designed for classification purposes to include patients in clinical trials and not for diagnosis, limiting their clinical applicability [118].

### 8.2. 2022 ACR/EULAR Classification Criteria

The updated 2022 classification criteria address many limitations of previous versions while incorporating modern diagnostic modalities [64]. The new criteria require age ≥ 50 years as absolute requirement plus cumulative score ≥ 6 points from weighted clinical, laboratory, and imaging features [64].

Key innovations include incorporation of imaging findings (positive temporal artery biopsy or halo sign +5 points), recognition of large vessel involvement (bilateral axillary involvement +2 points, FDG-PET activity throughout aorta +2 points), and expanded clinical features, including morning stiffness and scalp tenderness [64]. Validation studies demonstrate an area under the curve of 0.91 with 87% sensitivity and 95% specificity [64].

Despite significant advances, the 2022 ACR/EULAR criteria have important limitations that affect clinical implementation. The criteria’s incorporation of imaging findings, while improving sensitivity for large vessel disease, creates dependency on imaging availability and expertise that varies globally. In settings without access to vascular ultrasound or FDG-PET/CT, the criteria may perform less optimally than in resource-rich environments where they were primarily validated.

The weighted point-based system, while statistically more sophisticated than the simple 3-of-5 approach of the 1990 criteria, introduces practical challenges. Clinicians must calculate cumulative scores incorporating diverse elements with different point values, which may be cumbersome in busy clinical settings compared to dichotomous criteria. Furthermore, the requirement for age ≥ 50 years as an absolute inclusion criterion means that the criteria cannot classify younger patients with GCA-like presentations, though such cases, while rare, do occur.

An additional limitation is that classification criteria are designed to create homogeneous populations for clinical trials rather than to diagnose individual patients. The 2022 criteria were validated against final clinical diagnosis, but “gold standard” diagnostic criteria for GCA do not exist, potentially leading to circular reasoning. Patients with atypical presentations or early disease may not fulfill classification criteria, but, nonetheless, require treatment. This highlights the importance of not using them for diagnosis.

Finally, the criteria assign equal weight to halo sign and positive temporal artery biopsy (+5 points each), yet these tests have different performance characteristics depending on timing relative to glucocorticoid initiation, clinical phenotype, and technical quality. This simplified approach may not reflect the nuanced diagnostic reasoning required in complex cases.

### 8.3. Side-By Side Comparison of the 1990 ACR and the 2022 ACR/EULAR Classification Criteria for GCA

The 2022 ACR/EULAR classification criteria represent a major update to the 1990 criteria, primarily by integrating imaging studies and moving to a weighted point-based system. The new 2022 criteria aim to improve sensitivity and more accurately classify patients, particularly those with large vessel-GCA (LV-GCA) that do not involve the temporal arteries (Table 1).

The 1990 ACR criteria were validated against other forms of vasculitis, not against non-inflammatory conditions, which partially explains the performance differences in real-world clinical settings where the differential diagnosis is broader.

The new criteria were developed to address the limitations of the 1990 criteria, which had low sensitivity, especially for patients who primarily had large vessel involvement without typical cranial symptoms.

The key changes are listed below:

1. **Inclusion of imaging:** The most significant change is the incorporation of imaging—specifically ultrasound (halo sign) and FDG-PET/CT—allowing for the classification of patients based on objective non-invasive evidence.
2. **Increased weight for key findings:** Highly specific findings like a positive biopsy/halo sign (+5 points) and sudden visual loss (+3 points) are weighted more heavily, reflecting their high diagnostic value.
3. **Expanded clinical criteria:** Symptoms like PMR (morning stiffness) and scalp tenderness are formally included as weighted criteria, improving classification in the early or non-classic stages of the disease.

## 9. Differential Diagnostic Challenges

### 9.1. Mimicking Conditions

Different conditions share clinical features with GCA, and we call them mimickers. Most mimickers are infections, malignancies, atherosclerosis, and other vasculitides. Some of them can be easily distinguished through specific diagnostic tests, e.g., infectious etiologies identifiable through cultures or serology, or some malignancies that can be diagnosed by pathology, imaging, and/or laboratory testing. On occasions, the distinction is more challenging and requires a more comprehensive evaluation. A differential diagnosis should thoroughly rule out all the possible mimickers before diagnosing and treating GCA, especially in those patients without pathological confirmation.

The TAB, despite its limitations discussed in Section 7, maintains importance as a diagnostic tool, particularly when imaging results are inconclusive [120]. From my point of view, the importance of having a positive temporal artery biopsy lies in the confirmation of GCA avoiding the need for further evaluation. If the patient does not repond or relapses during treatment, we should pursue other treatment modalities for GCA or investigate the treatment compliance. In contrast, if the patient has had no biopsy done or if it has been negative, in case of lack of response to treatment, it may be necessary to perform additional testing to rule out other possible alternative diagnosis.

Infectious causes, including bacterial endocarditis, septicemia with mycotic aneurysms, syphilis, mycobacterial infections, and even viral infections, require exclusion through appropriate microbiological testing [121,122,123].

Malignancies may manifest with constitutional symptoms, elevated inflammatory markers, and vascular involvement, as in patients with GCA. Therefore, cases of suspected GCA necessitate comprehensive evaluation, including age-appropriate cancer screening [121,122].

Primary central nervous system vasculitis can mimic GCA with headache and neurological symptoms but typically affects younger patients and involves smaller intracranial vessels [124,125]. Other systemic vasculitides, including antineutrophil cytoplasmic antibody (ANCA)-associated vasculitis [126], Takayasu arteritis [127], and secondary vasculitis associated with connective tissue diseases [128] or infections [123], require differentiation through specific serological testing, imaging patterns, demographic characteristics, additional clinical manifestations, and biopsy findings [129]. In addition, complicating the picture even more, GCA may coexist with other types of systemic vasculitis [130]. Atherosclerotic disease with secondary inflammation may simulate large vessel involvement but typically demonstrates different distribution patterns and risk factor profiles [59,129].

Misdiagnosing GCA may have severe consequences [131]. It is important to remember that diagnostic criteria do not exist, and the classification criteria have been developed to decide inclusion of the patients in clinical trials; they often fail to differentiate mimics from true GCA [64,118,119]. Clinician vigilance and expertise, biopsy for pathology confirmation whenever possible, and fast improving imaging tools are critical for ruling out or confirming GCA and should be used widely used in the setting of careful clinical judgement and consideration of all possible alternative diagnosis. Diagnostic delays may result in severe outcomes, and patients who are misdiagnosed with GCA are at serious risk of suffering severe adverse effects from high doses of prolonged glucocorticoids therapy. Reducing diagnostic delay is essential for improving patient survival in severe mimic cases [131].

A systematic approach to excluding mimickers includes the following. (1) Comprehensive infectious workup in patients with fever or atypical features (blood cultures, HIV, syphilis, tuberculosis screening, viral serologies based on exposure history, coccidiosis, histoplasmosis, or Lyme disease depending on geographical location). (2) ANCA serology and urinalysis in all suspected GCA cases. (3) Age-appropriate cancer screening and consideration of PET/CT (which can identify both GCA and malignancy). (4) Assessment for features atypical for GCA that should prompt consideration of alternative diagnoses (age < 50 years, lack of cranial symptoms with isolated systemic features, neurological deficits, renal involvement, pulmonary infiltrates). (5) Temporal artery biopsy in diagnostically uncertain cases, or biopsy of other affected organs, as specific histopathological patterns (infectious organisms, malignant cells) can identify mimickers (Table 2).

### 9.2. Age-Related Diagnostic Considerations

The elderly population affected by GCA frequently presents diagnostic challenges due to multiple comorbidities and frequent atypical presentations [132]. Age-related changes, including presbyopia, cataracts, and chronic pain conditions, may mask or mimic GCA symptoms [133,134]. Polypharmacy and medication interactions may complicate laboratory interpretation and treatment decisions [135].

Cognitive impairment in elderly patients may limit symptom reporting accuracy and adherence to diagnostic procedures [136]. Healthcare providers must maintain a high index of suspicion while carefully evaluating competing diagnoses in this vulnerable population [137].

## 10. Treatment Implications and Monitoring

### 10.1. Glucocorticoid Therapy

High-dose glucocorticoids remain the cornerstone of GCA treatment. Optimal initial glucocorticoid dosing remains debated. Traditional regimens used prednisolone 40–60 mg daily for uncomplicated cranial disease, but recent evidence suggests that lower initial doses (30–40 mg daily) may provide equivalent efficacy with reduced toxicity in selected patients [138]. Patients with visual involvement require higher doses, with most guidelines recommending intravenous methylprednisolone 500–1000 mg daily for 3 days followed by high-dose oral therapy [139]. Treatment should be initiated immediately upon clinical suspicion without waiting for confirmatory testing, as delays beyond 48 h may result in irreversible complications, especially permanent visual loss [140].

Tapering strategies significantly impact outcomes. Rapid tapering (reaching 10 mg daily by 3 months) increases relapse risk compared to more gradual approaches. The GCA Actemra (GiACTA) trial’s 26-week taper (from 40 mg to zero) demonstrated feasibility in tocilizumab-treated patients but resulted in high relapse rates (86%) in placebo-treated patients [141]. The optimal tapering schedule remains debated, with EULAR recommendations suggesting reduction to 15–20 mg daily within 2–3 months and <5 mg daily by one year [142,143]. Current evidence supports individualized tapering based on symptoms, inflammatory markers, and imaging findings, with slower tapers (maintaining 10–15 mg daily for 6–12 months) in high-risk patients, including those with large vessel involvement, cranial ischemic complications, or relapsing disease [144,145]. However, relapse rates of 40–75% during tapering necessitate careful monitoring and individualized approaches [146]. Prolonged glucocorticoid exposure results in significant toxicity in >80% of patients, including posterior subcapsular cataracts (41%), bone fractures (38%), infections (31%), and hypertension (22%), among others [147].

### 10.2. Steroid-Sparing Agents

Tocilizumab represents the first Food and Drug Administration (FDA)-approved steroid-sparing agent for GCA, demonstrating significant efficacy in the GiACTA trial with sustained remission rates of 56% (weekly dosing) and 53% (every other week) compared to 14–18% with placebo at 52 weeks [141]. The drug provides substantial glucocorticoid-sparing effects and improved health-related quality of life [141].

Long-term tocilizumab studies reveal relapse rates of approximately 50% following discontinuation, suggesting a potential need for extended treatment in selected patients [148]. Cost considerations, side effects such as liver damage, worsening hyperlipidemia or increased infection risks, and the lack of universal patient response limit widespread accessibility, highlighting need for additional therapeutic options [149].

Upadacitinib was FDA-approved for the treatment of GCA in November 2024 based on the results of the SELECT-GCA clinical trial. The trial demonstrated that 15 mg daily of upadacitinib in combination with a glucocorticoid taper achieved a sustained remission rate of 46% compared to 29% in the placebo group, with a safety profile consistent with other approved indications [150].

Meta-analysis of individual patient data suggests that methotrexate 15–25 mg weekly provides modest reduction in relapse risk (RR 0.65–0.85) and cumulative glucocorticoid exposure (mean reduction 842 mg over first year), though these benefits are substantially smaller than those seen with tocilizumab or upadacitinib [151,152,153,154].

Practical considerations for methotrexate use include dosing typically 15–20 mg weekly (oral or subcutaneous), mandatory folic acid supplementation, baseline assessment and monitoring of liver and renal function and blood counts, and awareness that beneficial effects may not become apparent until 12–16 weeks of therapy. The modest benefit–risk profile suggests that methotrexate may be most appropriate for patients unable to access or tolerate biologics, or as adjunctive therapy in refractory cases.

Leflunomide shows promise in recent studies, with relapse reduction in over 75% of patients and favorable safety profile [155].

#### Critical Appraisal of Biologic Evidence

Tocilizumab’s efficacy is supported by the high-quality GiACTA trial (*n* = 251, randomized, double-blind) which demonstrated sustained remission in 56% (weekly dosing) vs. 14% (placebo) at 52 weeks. However, critical analysis reveals important limitations: (1) after tocilizumab discontinuation at 52 weeks, approximately 50% of patients relapsed within the subsequent year, raising questions about optimal treatment duration; (2) the trial excluded patients with recent visual loss, limiting generalizability to high-risk patients; (3) cost (approximately $30,000–40,000 USD annually) limits accessibility; and (4) while glucocorticoid-sparing effects are substantial, patients still received significant cumulative glucocorticoid exposure (1862 mg in weekly tocilizumab arm vs. 3296 mg in placebo arm) [141,156].

Upadacitinib approval is based on the SELECT-GCA trial (*n* = 428), which showed sustained remission in 46% vs. 29% (placebo) with similar safety to other indications [150]. Critical considerations include the following: (1) shorter follow-up (52 weeks) than available for tocilizumab (multiyear data available); (2) the 17-percentage-point difference, while statistically significant, represents modest absolute benefit; (3) the trial used a shorter glucocorticoid taper (26 weeks) compared to typical clinical practice; (4) as an oral medication, upadacitinib may offer convenience advantages over subcutaneous tocilizumab, but requires consideration of JAK-inhibitor class warnings (infections, malignancy, cardiovascular events) established in other diseases. While upadacitinib demonstrated a statistically significant benefit (46% vs. 29%, absolute difference 17 percentage points), this is notably more modest than tocilizumab’s performance in GiACTA (56% vs. 14%, absolute difference 42 percentage points), suggesting that tocilizumab remains the preferred biologic agent when glucocorticoid-sparing therapy is indicated.

Head-to-head trials comparing tocilizumab and upadacitinib are lacking, and biomarkers to guide selection between these agents are not available. Treatment selection currently relies on factors including patient preference for administration route, comorbidities, cost/insurance coverage, and physician experience.

### 10.3. Emerging Therapeutic Targets

**Figure 4:**
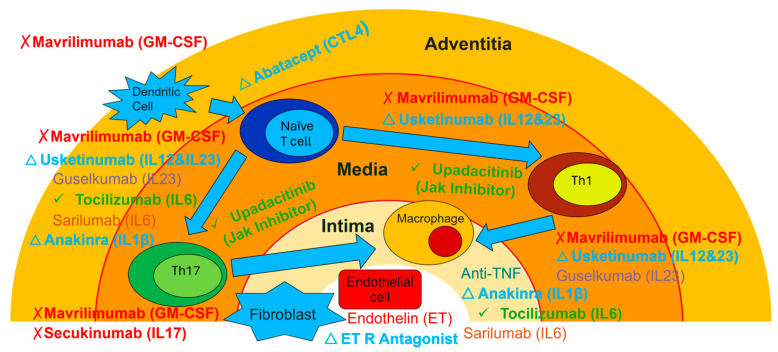
**Therapeutic targets in GCA according to pathogenesis.** This schematic illustrates the inflammatory cascade across the three layers of the arterial wall, the adventitia, media, and intima, and identifies the specific points of intervention for current and emerging biologic therapies. **In the adventitia,** the process is initiated by DCs which activate naïve T cells. This costimulation is targeted by abatacept (cytotoxic T-lymphocyte associated protein 4 [CTLA4]-Ig). **In the media,** activated T cells differentiate into Th1 and Th17 subsets. GM-CSF (targeted by mavrilimumab) and IL-12/23 (targeted by ustekinumab) drive this differentiation. JAK inhibitors act intracellularly to block these signaling pathways. **In the intima,** effector cells like macrophages and fibroblasts accumulate, leading to IL-6 production (targeted by tocilizumab/sarilumab) and TNF release. Endothelial cells release endothelin (ET), which promotes vascular remodeling and narrowing, countered by ET receptor antagonists. Agents are color-coded by regulatory/development status: green checkmark (✓)—FDA-approved agents (tocilizumab, upadacitinib); red X (✗)—Phase III failed (secukinumab) or development-terminated agents (mavrilimumab); blue triangle (△)—investigational agents (abatacept, ustekinumab, anakinra, endothelin receptor antagonists). **Abbreviations:** CTL4, Cytotoxic T-Lymphocyte Associated Protein 4; ET/ET R, endothelin/endothelin receptor, GM-CSF, Granulocyte-Macrophage Colony-Stimulating Factor; IL, interleukin; JAK, Janus Kinase; Th1, T Helper Type 1 cell; Th17, T Helper Type 17 cell; TNF, tumor necrosis factor.

Multiple novel therapeutic approaches are under investigation, reflecting improved understanding of GCA pathophysiology [114,157] (Figure 4).

IL-17 inhibition with secukinumab demonstrated encouraging results in Phase II trials, with sustained remission rates of 59.3% compared to 8.0% with placebo [158]. However, The Phase III GCAptAIN study (NCT04930094), which evaluated secukinumab in adults with newly diagnosed or relapsing GCA did not meet its primary endpoint of sustained remission at week 52 according to results announced by Novartis in 2024 (full peer-reviewed results pending publication). The failure of the Phase III trial to confirm Phase II results suggests that the Phase II findings may represent a false positive, and IL-17 inhibition does not appear to be an effective therapeutic target for GCA based on current evidence. Publication of the complete Phase III dataset will be important for understanding why the promising Phase II results were not reproduced.

GM-CSF receptor blockade with mavrilimumab targets multiple pathogenic pathways and showed significant flare reduction in Phase II studies [9]. In early 2024, Kiniksa terminated its licensing agreement for mavrilimumab and discontinued its development for GCA to pivot to a cardiovascular-focused pipeline, effectively halting the planned Phase III trial. This termination prevented definitive evaluation of the GM-CSF pathway in GCA and raises questions about the clinical translational potential of this mechanism in GCA management.

As some of these are recently completed trials, we note where information is derived from company announcements pending full peer-reviewed publication.

Additional investigational agents include IL-1 receptor antagonists (anakinra), IL-23 inhibitors (guselkumab), and T cell costimulation blockers (abatacept) [114]. These diverse mechanisms reflect the complex pathophysiology of GCA and potential for personalized therapeutic approaches based on individual disease phenotypes [159].

### 10.4. Long-Term Disease Management

Treatment duration varies considerably among patients, with meta-analysis revealing that 89.7% remain on glucocorticoids at one year, 75.2% at two years, and 44.3% at five years. Mean glucocorticoid doses at these timepoints are 9.1 mg/day and 7.8 mg/day, respectively [160].

### 10.5. Predictors of Disease Relapse

Disease relapse occurs in 30–50% of patients during treatment tapering, with higher rates in younger patients, females, and those with large vessel involvement [161].

Identifying patients at high relapse risk could enable personalized monitoring and treatment strategies. Established predictors of increased relapse risk include the following:

1. Clinical factors:
  - Large vessel involvement at diagnosis.
  - Younger age at diagnosis (<60–65 years).
  - Female sex (modest association.
  - PMR symptoms at diagnosis.
  - History of prior relapse.
  - Prolonged disease duration and treatment.
2. Laboratory/imaging markers:
  - Persistently elevated or slow-to-normalize inflammatory markers during initial treatment.
  - Persistent vascular inflammation on FDG-PET or ultrasound despite clinical remission.
  - Thrombocytosis at diagnosis or during follow-up.
3. Treatment-related factors:
  - Rapid glucocorticoid tapering (reduction >10 mg/month).
  - Glucocorticoid dose <10–15 mg daily at time of relapse consideration.
  - Non-adherence to treatment.

Machine learning algorithms incorporating multiple clinical and laboratory parameters have demonstrated 71–76% accuracy in predicting relapse, superior to individual risk factors, though these require prospective validation before clinical implementation. The development and validation of composite risk scores could enable stratified approaches with intensive monitoring and preemptive treatment adjustments for high-risk patients [161].

## 11. Prognosis and Long-Term Outcomes

### 11.1. Visual Outcomes

Visual recovery following GCA-related vision loss remains disappointingly limited, with only 15% of patients showing improvement in visual acuity within the first month, and 5% demonstrating corresponding visual field improvement [162]. Visual deterioration occurs in approximately 27% of eyes within the first week despite high-dose intravenous glucocorticoids, with greatest risk in the first six days [163].

The irreversible nature of most visual complications emphasizes the critical importance of prevention through early recognition and treatment [164]. Recent studies suggest that 13.7% of GCA patients experience vision loss, which rarely recovers and is statistically more likely with advancing age [36].

### 11.2. Cardiovascular and Aortic Complications

GCA patients demonstrate significantly increased risk of aortic aneurysm development, with five-year risk of 3.6% compared to 1.8% in controls. Thoracic aortic aneurysms occur in 2.2% versus 1.0% of controls, while abdominal aortic aneurysms affect 1.8% versus 0.8% [4]. These complications may develop years after initial diagnosis and require long-term surveillance [165].

Aortic dissection represents a rare but life-threatening complication, occurring in approximately 1% of patients [166]. Symptomatic aortitis at diagnosis, characterized by chest or abdominal pain, identifies patients at particularly high risk for subsequent aortic complications with hazard ratio of 6.64 [167,168]. However, overall survival may not differ significantly from age-matched populations, particularly beyond the first five years following diagnosis [169].

### 11.3. Prevention and Mitigation of GCA-Related and Treatment-Related Complications

Given that >80% of GCA patients experience glucocorticoid-related toxicity, proactive prevention strategies are essential.

All patients initiating glucocorticoid therapy should undergo baseline bone density assessment and receive calcium (1200–1500 mg daily) and vitamin D (800–1000 IU daily) supplementation, with bisphosphonate therapy for those with osteoporosis or high fracture risk [170,171,172].

Baseline screening and regular monitoring for diabetes and hypertension are critical, with early intervention when indicated.

Vaccinations should be updated before initiating immunosuppression (pneumococcal, influenza, zoster), and *Pneumocystis jirovecii* pneumonia (PJP) prophylaxis considered in patients receiving >20 mg prednisone daily for >1 month [173,174].

Annual ophthalmologic examination for cataracts and glaucoma is recommended. Given the 5-year aortic aneurysm risk of 3.6% (nearly double that of controls), systematic vascular surveillance is warranted. Baseline chest imaging to assess the thoracic aorta and consideration of periodic imaging (every 2–3 years) with CTA, MRA, or ultrasound for aortic assessment is recommended [175,176,177]. More frequent monitoring (annually) should be considered in patients with symptomatic aortitis at diagnosis, extensive large vessel involvement, or persistent vascular inflammation on imaging [175,176,177].

Emergency treatment protocols should be established for any new visual symptoms, with patient education regarding warning signs (amaurosis fugax, diplopia, visual field defects) [178,179].

Aspirin therapy (81–100 mg daily) may reduce cranial ischemic event risk, though evidence is not definitive [178,179].

Regular assessment of quality of life, physical therapy to maintain function, early introduction of steroid-sparing agents in high-risk patients, and screening for depression (affecting 20–30% of GCA patients) optimize long-term outcomes [180,181].

These preventive strategies require systematic implementation through structured care pathways [182].

## 12. Future Directions and Emerging Technologies

### 12.1. Artificial Intelligence (AI) and Machine Learning

AI applications in GCA diagnosis and management represent rapidly evolving areas with significant potential [161,183,184]. Machine learning algorithms demonstrate effectiveness in predicting GCA diagnosis and relapse risk, with random forest models achieving 71–76% accuracy using clinical and laboratory data [185,186,187].

Deep learning approaches for ultrasound image analysis show promise for automated halo sign detection and diagnostic support [183,184]. Convolutional neural networks applied to temporal artery biopsy specimens may enhance histopathological interpretation and reduce inter-observer variability [188].

Point-of-care ultrasound (POCUS) combined with AI-driven diagnostics could revolutionize GCA diagnosis, particularly in emergency and primary care settings [189]. Such systems could provide rapid, on-the-spot diagnosis and potentially prevent up to 500,000 cases of visual impairment projected by 2050 [190].

### 12.2. Advanced Imaging Technologies

Novel imaging techniques continue to expand diagnostic capabilities and disease understanding [191]. Contrast-enhanced ultrasound, shear wave elastography, and ultrasound biomicroscopy offer enhanced tissue characterization and may improve diagnostic accuracy [192].

PET-MRI integration combines the inflammatory detection capabilities of PET with superior anatomical resolution of MRI. Novel PET tracers beyond FDG may provide more specific inflammatory targeting and reduced background uptake [193].

Black blood MRI techniques and orbital MRI show particular promise for detecting cranial vessel involvement and ophthalmic complications. These advances may enable earlier detection of subclinical disease and better monitoring of treatment response [194].

### 12.3. Personalized Medicine Approaches

The recognition of distinct GCA phenotypes suggests potential for personalized diagnostic and therapeutic strategies. Evidence increasingly supports phenotype-based treatment stratification: (1) cranial-predominant GCA may be optimally managed with shorter treatment courses (18–24 months); (2) large vessel-predominant GCA likely requires longer treatment duration (>2–3 years) with routine imaging surveillance given higher relapse rates; and (3) PMR-overlap phenotype requires clarification regarding optimal management intensity [175,195].

Emerging evidence suggests that biomarker-guided therapeutic selection may improve outcomes. High IL-6 levels at diagnosis predict better tocilizumab response in some studies [196]. However, despite IL-6 having been detected in temporal arteritis, and the efficacy of IL-6-inbition treatments, the diagnostic value of serum IL-6 for GCA is limited. Although IL-6 measurement is increasingly available in many laboratories, it is likely to have similar limitations to the analysis of ESR and CRP despite much higher costs, which limits its use in clinical practice [197]. Imaging-detected persistent vascular inflammation during clinical remission identifies high relapse risk patients who may benefit from treatment intensification [198]. Pharmacogenomic applications show promise: polymorphisms in glucocorticoid receptor genes (NR3C1, HSD11B1, ABCB1) influence efficacy and toxicity [199], while variants in IL-6 pathway genes may predict tocilizumab response and [200,201].

Integration of clinical, laboratory, and imaging data into composite risk scores could enable precision medicine, including diagnostic risk scores, relapse risk scores, and complication risk scores [202]. Machine learning algorithms have demonstrated 71–76% accuracy in predicting relapse [187], though prospective validation is required before clinical implementation.

Translating personalized medicine to clinical reality requires validation of phenotype classifications in large multicenter cohorts, prospective biomarker validation studies with predefined treatment algorithms, clinical trials testing phenotype-specific protocols versus standard care, development of point-of-care assays, integration of prediction algorithms into electronic health records, and cost-effectiveness analyses [203,204].

## 13. Conclusions

Giant cell arteritis exemplifies the challenges of diagnosing and managing heterogeneous systemic vasculitis in an aging population. Three key developments have transformed the GCA landscape, and they define the path forward.

First, the diagnostic paradigm has shifted from biopsy-centric, criteria-based classification toward multimodal, probabilistic assessment. The integration of vascular imaging with clinical evaluation enables earlier diagnosis, particularly of large-vessel-predominant disease. The 2022 ACR/EULAR classification criteria formalize this approach, though accessibility challenges and the classification-versus-diagnosis distinction remain important limitations.

Second, therapeutic advances, particularly tocilizumab and upadacitinib approval, provide evidence-based alternatives to prolonged glucocorticoid monotherapy, yet important questions remain. The modest absolute benefits (46–56% sustained remission versus 14–29% with placebo) highlight the need for more effective therapies and biomarkers to guide therapeutic selection, optimal treatment duration, and strategies for preventing late complications.

Third, the evolution toward personalized medicine, supported by phenotypic classification, imaging-based risk stratification, and emerging predictive algorithms, offers potential to match treatment intensity to individual patient risk, though this requires prospective validation.

Key priorities for advancing GCA care include development of composite biomarker panels, comparative effectiveness trials of biologics and combination strategies, standardized implementation of fast-track diagnostic pathways, systematic aortic surveillance protocols, integration of AI-assisted diagnostic tools, and investigation of treatment de-escalation strategies.

Important limitations remain in the GCA literature, including heterogeneity in diagnostic criteria across studies, limited head-to-head imaging comparisons, predominantly Caucasian study populations limiting generalizability, and paucity of data on optimal treatment duration and safe discontinuation predictors.

GCA management has evolved from diagnostic uncertainty and treatment limited to glucocorticoids to an era of sophisticated imaging, mechanistically targeted therapeutics, and emerging precision medicine approaches. The ultimate goal, i.e., preventing irreversible complications while minimizing treatment toxicity through early, accurate diagnosis and personalized management, is increasingly achievable but requires sustained research investment and systematic quality improvement efforts.

## Figures and Tables

**Table 1:** Comparison of 1990 ACR and 2022 ACR/EULAR criteria.

Criteria	1990 ACR Criteria	2022 ACR/EULAR Criteria
**Number of items**	5 items.	10 items.
**Classification rule**	≥3 of 5 (equal weighting).	Score ≥ 6 points (weighted items).
**Age requirement**	One of 5 items.	Mandatory requirement.
**New additions**	N/A.	Morning stiffness, sudden vision loss, jaw claudication, CRP, temporal artery ultrasound, bilateral axillary involvement, FDG-PET.
**Sensitivity**	93.5% (vs. other vasculitides, original validation study); 53–80% (vs. non-vasculitic conditions, subsequent validation studies) *.	87%.
**Specificity**	80–91%.	95%.
**Strengths**	Simple, straightforward.	Better discrimination, incorporates imaging, improved large vessel detection.
**Limitations**	Poor for LV- GCA, no imaging criteria, outdated.	More complex, requires more investigations.
**Imaging modalities**	None (biopsy only).	Temporal artery ultrasound, FDG-PET.
**Best application**	Cranial GCA with systemic symptoms.	All GCA phenotypes (cranial, large vessel, constitutional).

* The original Hunder et al. 1990 [118] ACR validation study tested giant cell arteritis against other vasculitides only. The 53–80% specificity against non-vasculitic conditions is derived from subsequent validation studies [119,120].

**Table 2:** Distinguishing features of major GCA mimickers.

Mimicker	Shared Features with GCA	Distinguishing Features
**Infectious endocarditis**	Fever, elevated inflammatory markers, headache, anemia.	Blood cultures positive, heart murmur, cardiac imaging abnormalities, absence of temporal artery or large vessel inflammation on imaging.
**ANCA-associated vasculitis**	Constitutional symptoms, elevated inflammatory markers, vascular involvement.	Positive ANCA serology (anti-PR3 or anti-MPO), smaller vessel involvement (glomerulonephritis, pulmonary hemorrhage), different demographic (younger patients), nasal/sinus involvement common.
**Primary CNS vasculitis**	Headache, neurological symptoms, elevated inflammatory markers.	Neurological deficits, abnormal brain MRI with multiple lesions, CSF pleocytosis, angiographic abnormalities of intracranial vessels, negative temporal artery biopsy and extracranial imaging.
**Metastatic cancer**	Constitutional symptoms, elevated inflammatory markers, anemia, weight loss.	Identification of primary tumor or metastases on imaging, abnormal tumor markers, absence of arterial inflammation, lymphadenopathy.
**Atherosclerosis**	Large vessel involvement on imaging, vascular stenosis, old age.	Calcified plaques, eccentric rather than concentric vessel wall thickening, traditional cardiovascular risk factors, lower inflammatory marker elevations.

**Abbreviations:** ANCA, antineutrophil cytoplasmic antibody; CNS, central nervous system; CSF, cerebrospinal fluid; GCA, giant cell arteritis; MRI, magnetic resonance imaging; MPO, myeloperoxidase; PR3, proteinase 3.

## Data Availability

No new data were created or analyzed in this study. Data sharing is not applicable to this article.

## Abbreviations

The following abbreviations are used in this manuscript:

AAION	Arteritic anterior ischemic optic neuropathy
ACR	American College of Rheumatology
AI	Artificial intelligence
BAFF	B-cell activating factor
CD	Cluster of differentiation
CDUS	Color Doppler ultrasound
CTL4	Cytotoxic T-lymphocyte associated protein 4
CRP	C-reactive protein
CT	Computed tomography
CTA	Computed tomography angiography
DC	Dendritic Cell
ESR	Erythrocyte sedimentation rate
ET/ET R	Endothelin/Endothelin receptor
EULAR	European League Against Rheumatism
FDG	Fluorodeoxyglucose
FDG-PET	Fluorodeoxyglucose positron emission tomography
FR	Folate receptor
GiACTA	Giant cell arteritis Actemra trial
GM-CSF	Granulocyte macrophage colony-stimulating factor
HLA	Human leukocyte antigen
IL	Interleukin
i NOS	inducible nitric oxide synth2ase
JAK	Janus Kinase
IEL	Internal elastic lamina
IFN-γ	Interferon-γ
iNOS	inducible nitric oxide synthase
LV-GCA	Large vessel giant cell arteritis
MMP	Matrix metalloproteinase
MPO	Myeloperoxidase
MRA	Magnetic resonance angiography
MRI	Magnetic resonance imaging
PDGF	Platelet-derived growth factor
PET	Positron emission tomography
PMR	Polymyalgia rheumatica
POCUS	Point-of-care ultrasound
PR-3	Proteinase-3
SAA	Serum amyloid A
TAB	Temporal artery biopsy
TGF-β1	Transforming growth factor-β1
Th1	T Helper Type 1 cell
Th17	T Helper Type 17 cell
TNF	Tumor necrosis factor
VEGF	Vascular endothelial growth factor
